# Treadmill Exercise Can Modulate the Inflammatory Microenvironment and Promote Regeneration After Compressive Spinal Cord Injury in Female Mice

**DOI:** 10.1111/jcmm.71351

**Published:** 2026-10-13

**Authors:** Anne Caroline Rodrigues dos Santos Cavalcante, Renata Pereira Laurindo, Luiza dos Santos Heringer, Anna Lecticia Martinez Martinez Toledo, Gabriela Sena Souza, Gabriel Fidalgo Queiroz da Silva, Ana Carolina Campos dos Santos, Morgana Teixeira Lima Castelo Branco, Ana Maria Blanco Martinez, Suelen Adriani Marques

**Affiliations:** ^1^ Graduate Program in Pathological Anatomy Federal University of Rio de Janeiro Rio de Janeiro Brazil; ^2^ Department of Applied Nuclear Physics Federal University of Rio de Janeiro Rio de Janeiro Brazil; ^3^ Physics Department State University of Rio de Janeiro Rio de Janeiro Brazil; ^4^ Graduate Morphological Science Program Federal University of Rio de Janeiro Rio de Janeiro Brazil; ^5^ Institute of Biology, Neurobiology Department Federal Fluminense University Rio de Janeiro Brazil

**Keywords:** functional recovery, inflammatory microenvironment, morphological assessment and molecular assessment, spinal cord injury, treadmill training

## Abstract

Traumatic spinal cord injury (SCI) leads to severe sensorimotor impairments and reduced quality of life. Physical rehabilitation has emerged as a key therapeutic strategy to improve function and mitigate complications such as muscle atrophy and joint contractures, partly through modulation of the inflammatory microenvironment. This study investigated the effects of a structured exercise protocol on inflammation, neuroplasticity and sensorimotor recovery following compressive SCI in mice. Female C57BL/6 mice (8–12 weeks) were subjected to extradural spinal cord compression and divided into three groups: SHAM (laminectomy only), SCI (injury without treatment) and TMT (injury plus treadmill training). The TMT protocol began 7 days post‐injury and continued until 56 days, with sessions performed three times per week. Functional assessments were conducted weekly, followed by electroneuromyography at 56 days and biochemical and morphological analyses after euthanasia. The TMT group demonstrated significantly improved locomotor performance and nociceptive response compared to the SCI group. Electroneuromyography revealed higher compound muscle action potential amplitudes in TMT and SHAM groups (*p* < 0.001). Additionally, TMT reduced muscle atrophy (*p* < 0.05) and increased neuromuscular junction density. Bladder wall thickness in TMT animals resembled SHAM and differed from SCI (*p* < 0.001), suggesting reduced inflammation. At the molecular level, TMT increased anti‐inflammatory cytokines and reduced pro‐inflammatory markers, alongside enhanced M2 microglial polarisation, decreased astrocyte reactivity and reduced apoptosis. Imaging analyses confirmed reduced lesion spread and fibrosis. These findings indicate that phase‐adjusted exercise promotes functional recovery, neuromuscular plasticity and tissue preservation after SCI.

## 1 Introduction

Spinal cord injury (SCI) is a devastating neurological condition associated with high morbidity and mortality worldwide, resulting in profound physical, psychological and socioeconomic consequences [1]. Depending on the severity and level of injury, SCI leads to permanent or transient sensory, motor and autonomic impairments that markedly compromise functional independence and quality of life [2, 3, 4]. Locomotor dysfunction remains one of the most disabling clinical manifestations, reflecting the disruption of descending and ascending spinal pathways [5].

The pathophysiology of SCI involves two overlapping phases: the primary mechanical insult and the subsequent secondary injury cascade [2, 6, 7]. While the primary injury causes immediate tissue disruption, haemorrhage and axonal damage, the secondary phase is characterised by neuroinflammation, ischemia, oxidative stress, demyelination, apoptosis, glial activation and glial scar formation [8, 9]. Increasing evidence indicates that persistent neuroinflammation plays a central role in determining the balance between degeneration and repair after SCI. The inflammatory microenvironment directly regulates neuronal survival, precursor cell proliferation, axonal regeneration, remyelination and tissue remodelling, making immunomodulation an attractive therapeutic target [7, 10].

Among the available therapeutic approaches, physical rehabilitation remains one of the few interventions routinely implemented in clinical practice after SCI. Beyond improving motor performance, growing evidence suggests that exercise exerts neuroprotective effects by modulating inflammatory signalling pathways, regulating cytokine production, stimulating neurotrophic factor expression and promoting activity‐dependent neural plasticity [2, 11, 12, 13]. Nevertheless, the biological mechanisms linking exercise‐induced immunomodulation to structural preservation and functional recovery remain incompletely understood.

Experimental SCI models provide a controlled platform to investigate these mechanisms and optimise rehabilitation paradigms. Such models have demonstrated that exercise can enhance neuroplasticity, facilitate spinal circuit reorganisation, preserve neuromuscular junctions and attenuate muscle atrophy, although the cellular and molecular pathways responsible for these benefits have not been fully elucidated [14, 15, 16].

Although our group previously demonstrated that modulation of treadmill exercise volume improves functional recovery after compressive SCI [17], the biological mechanisms underlying these functional improvements remain largely unknown. Specifically, it is unclear whether exercise‐induced recovery is associated with sustained remodelling of the inflammatory microenvironment, modulation of resident microglial phenotype, attenuation of astrocytic reactivity, reduction of apoptosis, preservation of white matter integrity and maintenance of neuromuscular connectivity. Addressing these questions is essential to better understand how rehabilitation influences tissue repair beyond behavioural recovery.

Therefore, the aim of the present study was not merely to confirm the beneficial effects of treadmill exercise on locomotor recovery, but to investigate the cellular and molecular mechanisms underlying this response. Specifically, we evaluated whether a phase‐adjusted treadmill rehabilitation protocol promotes long‐term immunomodulation, microglial polarisation, astrocyte regulation, tissue preservation, neuromuscular plasticity and improved neural conduction following compressive SCI in mice.

## 2 Materials and Methods

### 2.1 Spinal Cord Surgery

The experimental procedures were approved by the Federal University of Rio de Janeiro Animal Care Committee (CEUA/UFRJ, protocol number 01200.001568/2013‐87). 52 Adult female mice (the choice of female donors was to reduce the possibility of genital injury due to the surgical procedure), aged 8–12 weeks, were divided into three groups: SHAM (laminectomy only), SCI (lesion without treatment) and TMT (lesion plus treadmill training). The sample size (*n* = 52 females) was estimated based on statistical parameters (*α* = 0.05; power = 80%) and the expected effect derived from previous spinal cord injury studies, ensuring the robustness of the results while adhering to the principle of reduction in animal use. The SCI and TMT groups were subjected to laminectomy followed by an extradural compressive SCI after being anaesthetised intraperitoneally with ketamine and xylazine (100 and 15 mg/kg, respectively). We performed the injury with a 30‐g vascular clip (Kent Scientific Corporation, Torrington, CT, USA) applied at the T9 level for 10 s. Injured animals received manual bladder expression twice daily until the return of bladder function. All the animals were kept in an animal facility with a light–dark cycle and free access to water and food. Only the animals that survived until the end of the lifespan (56 days) were included in the study.

### 2.2 Treadmill Training

Treadmill rehabilitation was performed using a motorised treadmill (Insight, Ribeirão Preto, SP, Brazil). Animals were trained at speeds ranging from 6 to 12 m/min, with intensity individually adjusted according to locomotor performance and tolerance throughout the experimental period. The rehabilitation protocol was based on our previous dose–response study [17], which demonstrated that exercise volume should be adapted according to the stage of injury to maximise functional recovery while minimising excessive physical stress.

Training was initiated at 7 days post‐injury (dpi), corresponding to the subacute phase of SCI and continued until 56 dpi. From 7 to 28 dpi, animals performed two 10‐min training sessions separated by a 10‐min rest interval. After 28 dpi, animals completed a single 10‐min session per training day. Sessions were conducted three times per week.

The training frequency was intentionally maintained at three weekly sessions to reproduce a clinically feasible rehabilitation strategy while avoiding excessive exercise during the early stages of tissue repair. Rather than maximising exercise volume, the protocol was designed to provide sufficient locomotor stimulation to promote activity‐dependent neuroplasticity while allowing adequate recovery between sessions. This strategy was selected based on our previous findings demonstrating that exercise dosage should be adjusted according to the temporal evolution of SCI rather than simply increasing training frequency [17].

### 2.3 Behavioural Analysis

Functional recovery was assessed using the Basso Mouse Scale (BMS) [18] and the Ladder Walking Test (LWT) [19]. Whenever applicable, behavioural evaluations were performed by investigators blinded to the experimental groups. BMS scores were obtained before surgery, 24 h after SCI to confirm injury severity and weekly thereafter until 56 days post‐injury (dpi). Hindlimb locomotion was independently evaluated, and the mean score of both hindlimbs was calculated for each animal (*n* = 5 per group).

The LWT was performed weekly from 28 to 56 dpi, when animals had recovered sufficient locomotor capacity to complete the task. Mice crossed a horizontal ladder with irregularly spaced rungs, whose configuration was periodically modified to minimise learning effects. Three trials per animal were recorded using a digital camera (30 frames/s), and the total and correct steps for each hindlimb were quantified and averaged.

### 2.4 Mechanical Sensitivity Assessment

Mechanical sensitivity was evaluated using an electronic von Frey anesthesiometer (Insight, Ribeirão Preto, Brazil). Animals were acclimated to the testing chambers for 15 min before each assessment. Paw withdrawal threshold (PWT) was determined by applying a polypropylene tip to the plantar surface of each hind paw until withdrawal occurred. Three consecutive measurements were obtained for each paw, and the mean value for each animal was used for statistical analysis (*n* = 5 per group).

### 2.5 Electroneuromyography Analysis

Electrophysiological evaluation was performed at 56 days post‐injury using a PowerLab 4/35 acquisition system (AD Instruments, Sydney, Australia) coupled to LabChart software. Animals were anaesthetised with ketamine (100 mg/kg) and xylazine (15 mg/kg), and a laminectomy was performed at the C0–C1 level to expose the cervical spinal cord for electrical stimulation. A bipolar stimulating electrode was positioned over the cervical spinal cord, and supramaximal electrical stimuli (10 mV) were delivered under identical acquisition parameters for all experimental groups. Compound muscle action potentials (CMAPs) were recorded from the ipsilateral gastrocnemius muscle using a monopolar recording electrode, while the reference electrode was placed in the dorsal subcutaneous tissue.

Five consecutive stimuli were applied to each animal, and CMAP amplitude and latency were measured. Mean values obtained from each animal were used for statistical analysis (*n* = 5 per group). All recordings were acquired using identical stimulation and recording settings to ensure consistency among experimental groups, allowing comparison of relative changes in motor conduction following SCI and treadmill rehabilitation.

### 2.6 Tissue Processing for Morphometric Analysis

After the end of survival time, a 1 mm segment of the spinal cord containing the epicentre of the lesion was dissected and post‐fixed, following the transmission electron microscopy (TEM) protocol. Briefly, the spinal cord segment from each animal was post‐fixed at room temperature for six hours in 1% osmium tetroxide containing 0.8% potassium ferrocyanide in 0.1M cacodylate buffer. Spinal cords were dehydrated in a graded series of acetone (30%, 50%, 70%, 80%, 90% and 100% for 10 min each), embedded overnight in Poly/Bed 812 resin (EMS, Houston, TX, USA) and polymerised for 48 h at 60°C. Semithin sections (500 μm) were cut on an ultramicrotome (RMC Boeckeler, Tucson, AZ, USA), collected on glass slides and stained with toluidine blue. The photomicrograph was obtained by an Axioskop 2 Plus microscope equipped with AxioVision version 4.5 (Zeiss, Germany).

### 2.7 Nerve Fibre Quantification and g‐Ratio Analysis

Digital images (1000× magnification) from semithin cross‐sections taken from the epicentre of the lesion (*n* = 5 for each group) [20] were used to quantify the number of preserved myelinated nerve fibres and to calculate the myelin, axon and nerve fibre areas and to calculate the g‐ratio (*n* = 5 for each group) using Image J software (Jandel Scientific). Six image fields per animal (three on each side of the spinal cord) were obtained in the anterior and lateral funiculus white matter areas. The g‐ratio was calculated as the ratio of the internal axonal diameter to the external diameter of the fibre.

### 2.8 Immunofluorescence Labeling

Immunofluorescence analyses were performed on transverse cryosections encompassing the lesion epicentre, as well as segments located approximately 1 mm rostral and 1 mm caudal to the injury site. The slides containing the spinal cord sections were blocked by endogenous biotin (with potassium permanganate for 10 min), washed (phosphate‐buffered saline, pH 7.4 twice for five minutes) and blocked to non‐specific sites [PBS‐0.3% Triton X‐100 plus 10% normal goat serum (NGS)] for one hour at room temperature. Then, the slides were incubated with the primary antibodies: rabbit polyclonal antibody TMEM 119 (1:100, HPA051870, Sigma), mouse monoclonal nitric oxide synthase antibody (1:100, sc‐7271, Santa Cruz), arginase 1 mouse monoclonal antibody (1:100, sl6arg, Invitrogen), active caspase 3 rabbit polyclonal antibody (1:100, AB3623, Merck), GFAP rabbit polyclonal antibody (1:500, G9269, Sigma), in the blocking solution for twenty‐four hours at room temperature. Negative controls were performed with the primary antibody omission for all reactions. Subsequently, the slides were thrice washed in PBS for five minutes each and then incubated with the secondary antibodies: Alexa Fluor 546 goat anti‐rabbit (1:500, A11035, Molecular Probes), Alexa Fluor 488 goat anti‐mouse (1:500, A11001, Molecular Probes), Alexa Fluor 488 goat anti‐mouse (1:500, A11006, Invitrogen), for two hours at room temperature. Then, the slides were washed and incubated for five minutes with nuclear marker 4,6‐diamino‐2‐phenylindole (DAPI, 20 mg/mL, Sigma).

For staining analysis (TMEM+arginase, TMEM+iNOS20, GFAP and cleaved caspase‐3), three spinal cord micrographs from the mice were captured (1 photo of the epicentre of the injury, one above the epicentre and one below the injury) using the confocal microscope (LEICA, TCS SPS) and Axiocam MRC camera with the Axiovison programme (200× magnification), standardising the same exposure time to capture all images. Immunofluorescence data were quantified using ImageJ software (Jandel Scientific, Corte Madera, CA, USA) by determining the integrated density for each section, giving *n* = 3/group. For each marker, images were acquired using identical microscope settings for all experimental groups. Quantitative analyses were performed under blinded conditions to minimise observer bias.

### 2.9 Analysis of the Inflammatory Microenvironment in the Spinal Cord

ELISA assays were used to analyse the cytokine profile 56 days after the injury. The fresh spinal cord (with approximately 2.5 mm) was collected from mice and maintained for 24 h in a humid atmosphere with 5% CO_2_ at 37°C in DMEM low medium (1000 μL) (Gibco, USA) supplemented with 10% fetal bovine serum (FBS). Pro‐inflammatory (IL‐1β, IL‐6 and TNFα) and anti‐inflammatory (IL‐4, IL‐10, IL‐13) cytokines were quantified in the spinal cord supernatant (*n* = 3/group) using the commercial kit from R&D Systems, following the established protocol. Briefly, 100 μL samples were added to each well in a 96‐well plate previously coated with antibodies, followed by incubation for 2 h at room temperature. Subsequently, 100 μL of working solution was added to each well, followed by an additional 1 h incubation at room temperature before adding the 100 μL substrate solution. The reaction was interrupted by a stop solution (50 μL), and the absorbance reading was performed at 450 nm.

The bladder wall was dissected and embedded in paraffin to evaluate systemic inflammatory response 56 days post‐injury. For this, the tissue was washed in 0.1M phosphate buffer, pH 7.4 and distilled water, dehydrated in increasing concentrations of alcohol (1‐h bath at dilution of 70%, 80% and 90%, and two 1‐h baths in the absolute alcohol), diaphanised in xylene (2 sequential baths for 1 h each), impregnated in paraffin (two paraffin baths at 56°C in an oven) and included, in a transverse position, in paraffin. The blocks were cut at 10 μm thickness using a microtome (45 Rotary microtome, Lipshaw MFG. Co), and the cross‐sections were stained by haematoxylin–eosin (HE) to visualise tissue morphology and perform the quantitative analysis. Three sections per animal were used for this analysis, with *n* = 3/group.

### 2.10 Soleus and Gastrocnemius Muscle Analysis

The soleus and medial gastrocnemius muscles were dissected and weighed on a precision scale after transcardiac perfusion with 4% paraformaldehyde and 2.5% glutaraldehyde to evaluate the loss of muscle mass due to atrophy, registering an average per group. Fixed muscles were cryoprotected (10%, 20% and 30% sucrose in 0.1M phosphate buffer pH 7.4, 24 h each), embedded in OCT (Tissue‐Tek, USA), frozen at −20°C and 60 μm thick longitudinal sections were cut in a cryostat (Leica CM 1850) to evaluate the neuromuscular junctions (*n* = 5/group). The slides were incubated in an oven at −57°C for 10 min, washed (PBS pH 7.4) twice for five minutes and blocked to non‐specific sites (PBS‐0.3% Triton‐X plus 10% NGS, at 1 h at room temperature). The sections were incubated with the α‐Bungarotoxin marker (1:200, B13423, Invitrogen) diluted in the blocking solution for 2 h, washed (PBS pH 7.4) four times for five minutes each and then mounted. Photomicrography was taken at 200× and 400× magnification using a confocal microscope (LEICA, TCS SPS). Motor endplates were quantified under a microscope using three serial sections per animal with a minimum distance of 400 μm to avoid counting twice the same motor endplate [21]. These results were correlated with electroneuromyography and functional data to assess neuromuscular plasticity.

### 2.11 Analysis by Microtomography and Scanning Electron Microscopy

Samples were prepared as described above in the tissue processing topic. Briefly, tissue was dehydrated by acetone solution and infiltrated in epon. After drying the tissue infiltrated in epon, microtomography was performed with a SkyScan 1275 micro‐CT scanner (Bruker) for three‐dimensional investigations of samples with high resolution, using previously optimised imaging conditions [5, 22]. The microtomography technique was conducted under specific conditions, using a voltage of 41 kV and a current of 200 μA. The exposure time was set at 55 ms to ensure that detailed images were obtained. The NRecon 1.7 software (Bruker) was used to reconstruct the images. The pixel size was set to 10.0 μm while the sample was rotated through 180° with an angular pitch of 0.2° to capture a comprehensive range of three‐dimensional information that is crucial to the proposed analysis.

Scanning electron microscopy (SEM) was used to verify the spinal cord topography after the survival time (one segment containing the epicentre of the lesion with 2 mm rostral and 2 mm caudal segments). After fixation by immersion, the samples were processed for TEM, positioned on a support containing double‐sided adhesive tape, sprayed with gold (FL‐9496 Balzers Union Coater), with the lesion side facing upwards and metalised using a metaliser (Denton Vacuum Desk II, United States of America). The samples were taken to a scanning electron microscope (EVO MA10—ZEISS) for analysis and obtention of electron micrographs.

### 2.12 Statistical Analysis

All analyses were performed using GraphPad Prism 6.0 software (Graph Pad Software, Boston, MA, USA). Data were analysed using one‐way or two‐way analysis of variance (ANOVA) or Student's *t*‐test followed by Tukey's or Bonferroni post‐tests. Data are expressed as mean ± SEM, and *p* < 0.05 was considered statistically significant.

## 3 Results

### 3.1 Treadmill Exercise Can Promote Locomotor and Sensory Functional Recovery

Locomotor recovery was first evaluated using the Basso Mouse Scale (BMS). As expected, SHAM animals maintained normal locomotor performance throughout the experimental period (9.0 ± 0.0). SCI mice exhibited persistent locomotor impairment and remained within the early stage of functional recovery during the 56‐day follow‐up. In contrast, treadmill rehabilitation significantly improved locomotor performance from 21 days post‐injury (dpi) onward, with higher BMS scores than untreated SCI animals (4.02 ± 0.45 vs. 1.48 ± 0.29, *p* < 0.001) (Figure 1A). By the end of the experimental period, treadmill‐trained animals reached the intermediate phase of locomotor recovery, indicating that rehabilitation promoted sustained functional improvement after compressive SCI.

**FIGURE 1:**
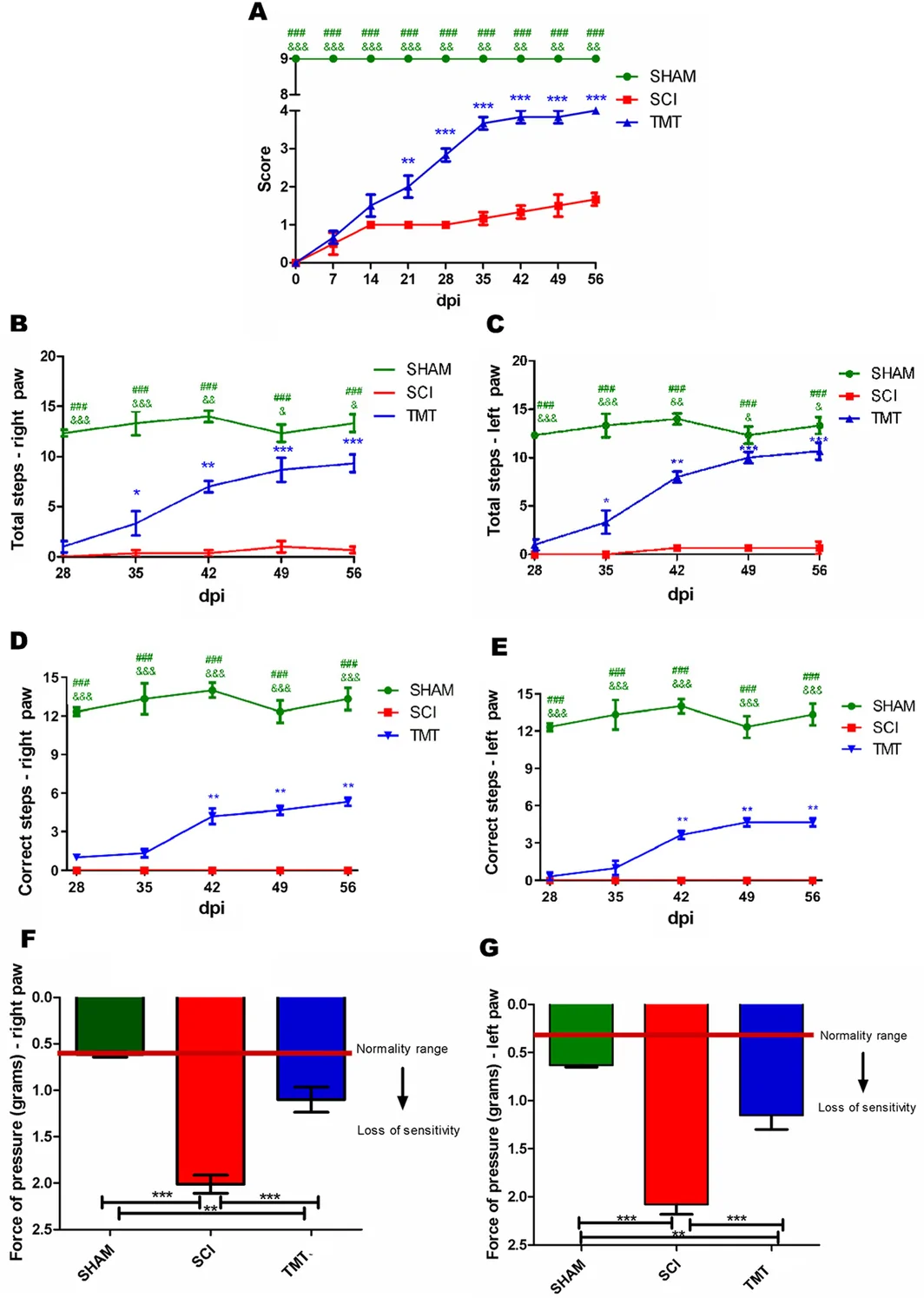
Treadmill exercise can promote locomotor and sensory functional recovery. (A) Line graph showing progressive BMS results over the studied survival time. For the entire survival time, the SHAM group obtained a statistically significant difference in relation to the SCI (#) and TMT (&) groups. From 21 days onwards, the TMT group showed a statistical difference in relation to the SCI group, maintaining this difference until the 56th day. (B, C) Data presented separately by paw (PD: Right paw (A and C); PE: Left paw (B and D)), due to evident asymmetry in mice after spinal cord injury. In relation to the total number of total steps taken, the TMT group showed a statistical difference in relation to the SCI group from 35 days post‐injury. (D, E) In relation to the number of correct steps, there was a significant difference between the injured groups, from the 42nd day onwards. SHAM group obtained a significant statistical difference in relation to SCI (#) and TMT (&). (F, G) Comparison of the evolution of mechanical sensitivity, on the 56th day, through the evaluation of the results of the digital analgesimeter (electronic Von Frey), data presented with the average between the right (a) and left (b) paws. Representation of pressure values (in grams), recorded by the analgesimeter at the moment of the reflex to withdraw the tested paw. The injured groups had an increased detection threshold when compared to SHAM. Although the SHAM group presented a lower threshold than the TMT group (*p* < 0.01), both the SHAM and TMT groups presented better results than the SCI (*p* < 0.001), with a statistically significant difference. Statistical analysis: Two‐way ANOVA and Tukey's post‐test. Values *p < 0.05; && and **p < 0.01, ###, &&& and ***p < 0.001; *n* = 5/group.

The Ladder Walking Test further confirmed the beneficial effects of treadmill rehabilitation on skilled locomotion. Although all injured animals were initially unable to perform the task, treadmill‐trained mice exhibited a significant increase in the total number of steps by 35 dpi compared with untreated SCI animals (*p* < 0.01). More importantly, the number of correct steps progressively increased throughout the experimental period, reaching significantly higher values at 56 dpi (*p* < 0.01). Despite not achieving SHAM performance, treadmill‐trained animals displayed markedly better motor coordination and paw placement accuracy than untreated SCI mice, indicating improved fine motor control (Figure 1B–D).

Mechanical sensitivity was evaluated using the electronic von Frey test. As expected, SCI markedly increased the mechanical withdrawal threshold, indicating sensory impairment after injury. Although all injured animals exhibited reduced sensitivity compared with SHAM controls, treadmill rehabilitation significantly attenuated this deficit. At 56 dpi, withdrawal thresholds in the TMT group were significantly lower than those of untreated SCI animals (*p* < 0.001) and approached SHAM values, suggesting partial preservation of sensory function following exercise (Figure 1E,F), averaging them. The values of the SHAM group (RP: 0.61 ± 0.02—LP: 0.62 ± 0.01) were used as the baseline. All injured groups showed a sensitivity loss, but the TMT group (RP: 1.10 ± 1.17; LP: 1.15 ± 1.12) exhibited better results than SCI (RP: 2.20 ± 0.65, LP: 2.01 ± 0.68, *p* < 0.001) on the 56th day, with results closer to the SHAM group.

Electrophysiological analysis demonstrated that treadmill rehabilitation preserved motor conduction after SCI. While untreated SCI animals exhibited a significant reduction in CMAP amplitude together with prolonged latency, treadmill‐trained mice showed electrophysiological parameters that were not significantly different from those of SHAM animals. Although the TMT group showed a decrease in the CMAP amplitude (1.18 ± 1 0.05) and an increase in the latency (0.0018 ± 0.21) there was no statistical difference compared to SHAM (Figure 2), while the SCI group presented the worst result in the CMAP amplitude (0.59 ± 0.79) and latency (0.0018 ± 0.21), compared to the other groups (SHAM 1.55 ± 0.33, *p* < 0.01 and TMT 1.18 ± 1 0.05, *p* < 0.05).

**FIGURE 2:**
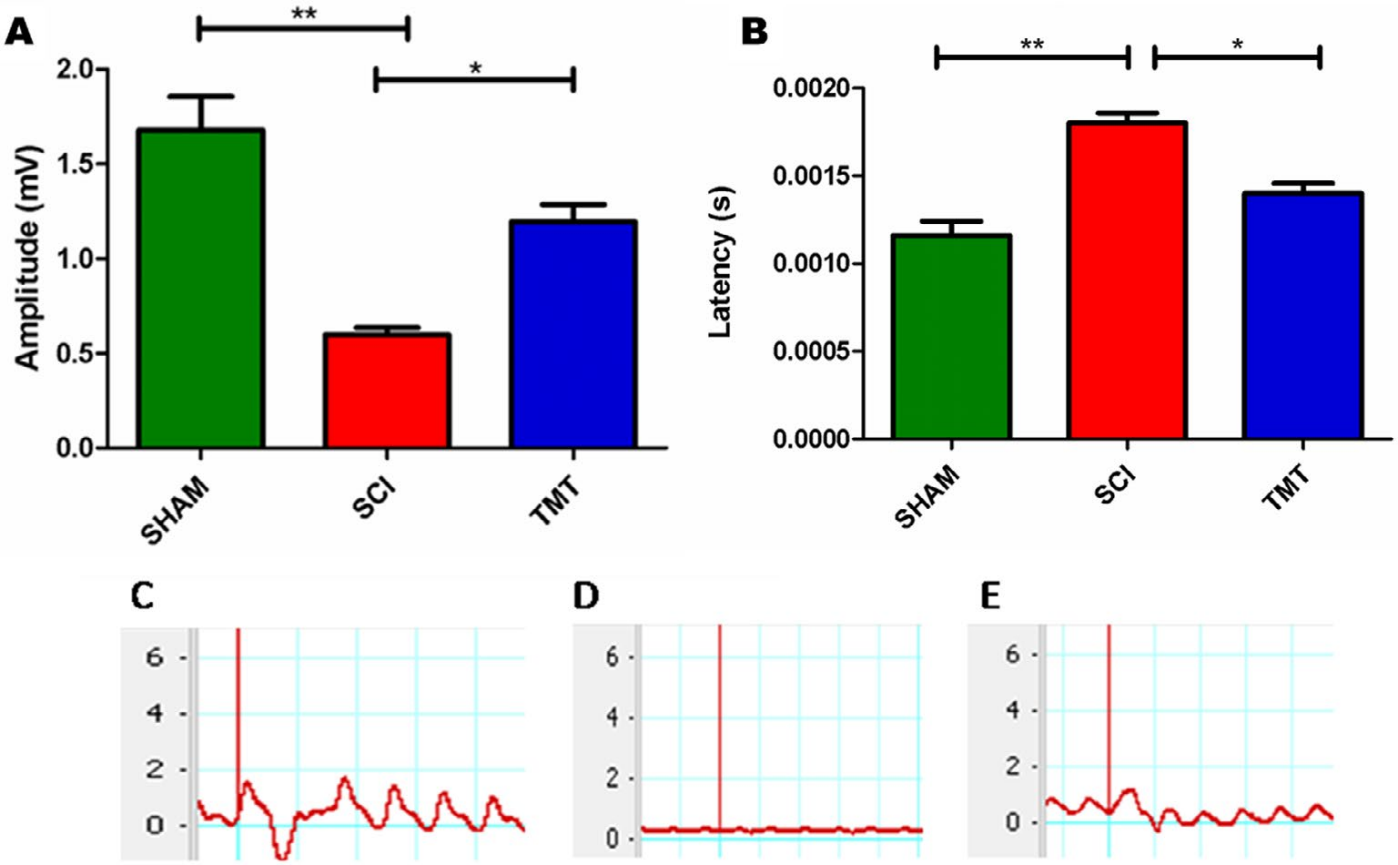
The amplitude and latency in electroneuromyography is better in the group trained after the injury. (A) The SHAM group showed a significant difference in relation to the injury‐only group, in terms of amplitude; however, there was no difference when compared to TMT. The TMT group performed better on the ENMG and showed a statistically significant difference compared to the SCI group. (B) Regarding electroneuromyography latency, all groups obtained a statistically significant difference in relation to the SCI group. These data demonstrate the effectiveness of the treatment protocol, with the TMT group trying values close to the normal standard. (C–E) Electroneuromyography tracings referring to the SHAM (C), SCI (D) and TMT (E) groups. Statistical analysis: One‐way ANOVA and Tukey's post‐test. *p < 0.05; **p < 0.01; *n* = 5/group.

### 3.2 Treadmill Rehabilitation Preserves Muscle Integrity and Neuromuscular Junction Morphology After SCI

Muscular atrophy (Figure 3) was assessed by the soleus and gastrocnemius weight to evaluate the impact of our exercise protocol on reinnervation and preservation of muscle mass. Although the muscles from the injured groups (SCI and TMT) showed a significant weight reduction compared to the SHAM (soleus: 0.017 ± 0.0549, *p* < 0,001; gastrocnemius: 0.0723 ± 0.3274, *p* < 0.001 and *p* < 0.01 to SCI and TMT, respectively), the TMT group showed less muscle mass reduction in both muscles (soleus 0.011 ± 1.078 and gastrocnemius 0.573 ± 0.0257, *p* < 0.05) compared to SCI (soleus 0.07 ± 0.0061 and gastrocnemius 0.0373 ± 0.0211). The TMT gastrocnemius muscles (*p* < 0.001) showed more preserved fibres than the soleus (*p* < 0.05) compared to the SHAM group (SCI and TMT), which is probably due to the influence of the exercise protocol used in the TMT group.

**FIGURE 3:**
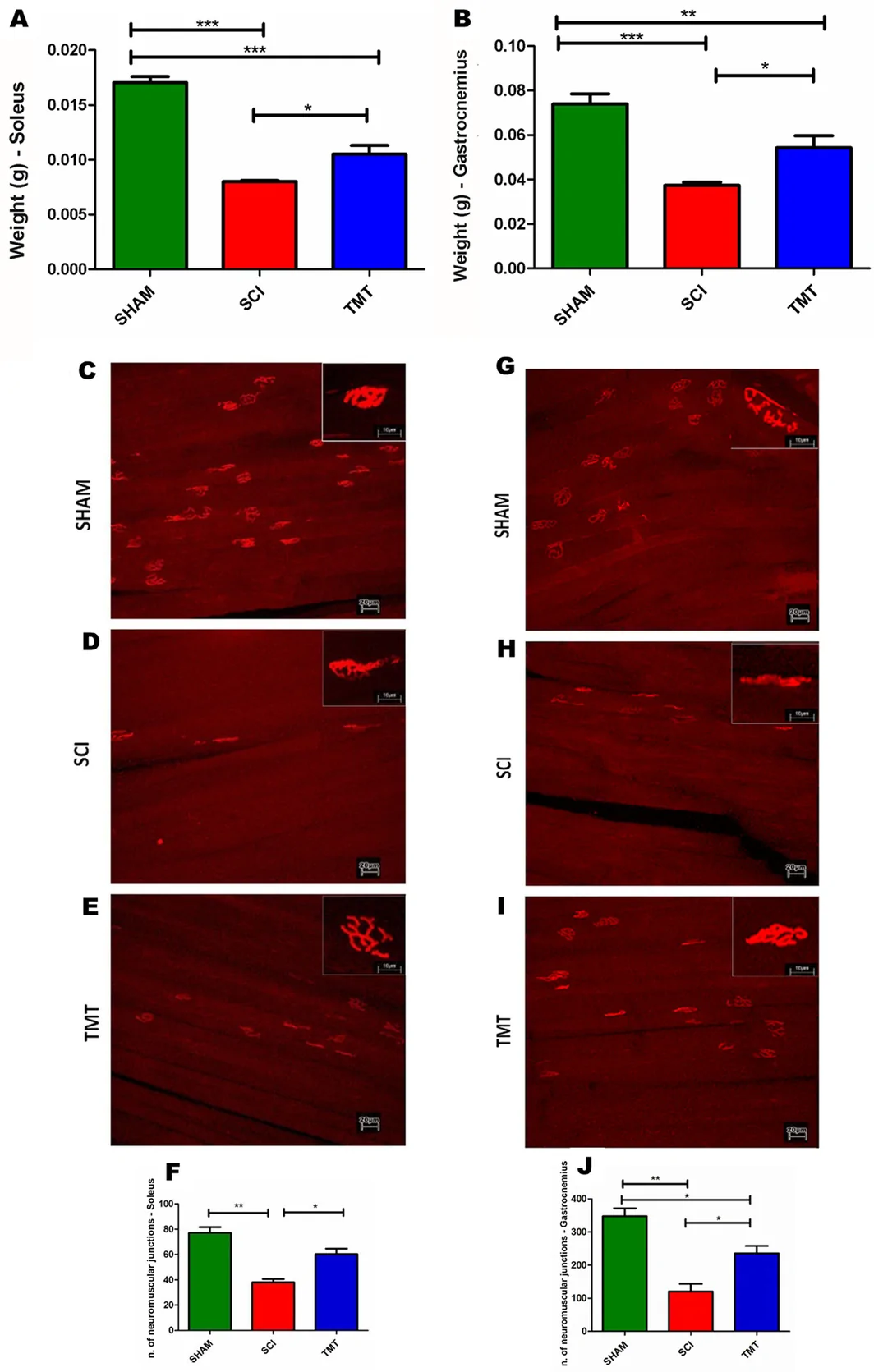
Treadmill exercise promotes prevention of atrophy and preservation of muscle function after spinal cord injury. (A) The SHAM group showed a significant difference when compared to the injured group in both muscle analyses. (B) The TMT group also showed a significant difference when compared to the SCI in the evaluation of the soleus and gastrocnemius. (C–E) and (G–I) Representation of markings from the SHAM, SCI and TMT groups for evaluation of motor endplates. Scale: 20 and 10 μm, respectively. Bar graphs representing the quantification of neuromuscular junctions in the soleus and gastrocnemius. For the soleus (F), we obtained a significant difference between the SHAM and TMT groups and the only injured group. When evaluating the gastrocnemius (J), the SHAM group demonstrated greater preservation of neuromuscular junctions, verifying differences between the control and injured groups. Furthermore, there was a difference between the SCI and TMT groups, with TMT having a greater number of junctions when compared to SCI. Statistical analysis: One‐way ANOVA and Tukey's post‐test. *p < 0.05; **p < 0.01; ***p < 0.001; *n* = 5/group.

The neuromuscular junctions were evaluated for α‐bungarotoxin labelling in the muscles to obtain qualitative (Figure 3C–E,G–I) and quantitative (Figure 3F,J) data. Motor plates' morphological preservation (maintaining the ‘pretzel’ shape) among the groups was assessed by α‐bungarotoxin labelling in the soleus and gastrocnemius muscles. The TMT group showed better preservation of neuromuscular junctions compared to the SCI (soleus: 72, *p* < 0.05, gastrocnemius: 261, *p* < 0.01), evidencing the exercise's effectiveness in maintaining neurotransmission and favouring functional return after injury.

### 3.3 Treadmill Rehabilitation Promotes an Anti‐Inflammatory Microenvironment and Preserves Spinal Cord Architecture After SCI

The pro‐inflammatory (TNF‐α, IL‐1β and IL‐6, Figure 4) and anti‐inflammatory cytokines (TGF‐β, IL‐10 and IL‐13, Figure 4) were measured by ELISA to analyse the spinal cord local inflammatory profile. The SHAM group values were considered the baseline and compared to the injured groups (SCI and TMT). The TMT group showed increased levels of anti‐inflammatory cytokines and lower expression of pro‐inflammatory cytokines, while the SCI group exhibited a clear pro‐inflammatory profile, with high levels of pro‐inflammatory cytokines, suggesting that the exercise can induce anti‐inflammatory modulation in the environment after spinal cord injury. Exercise, therefore, could favour polarisation about the inflammatory population towards an anti‐inflammatory profile.

**FIGURE 4:**
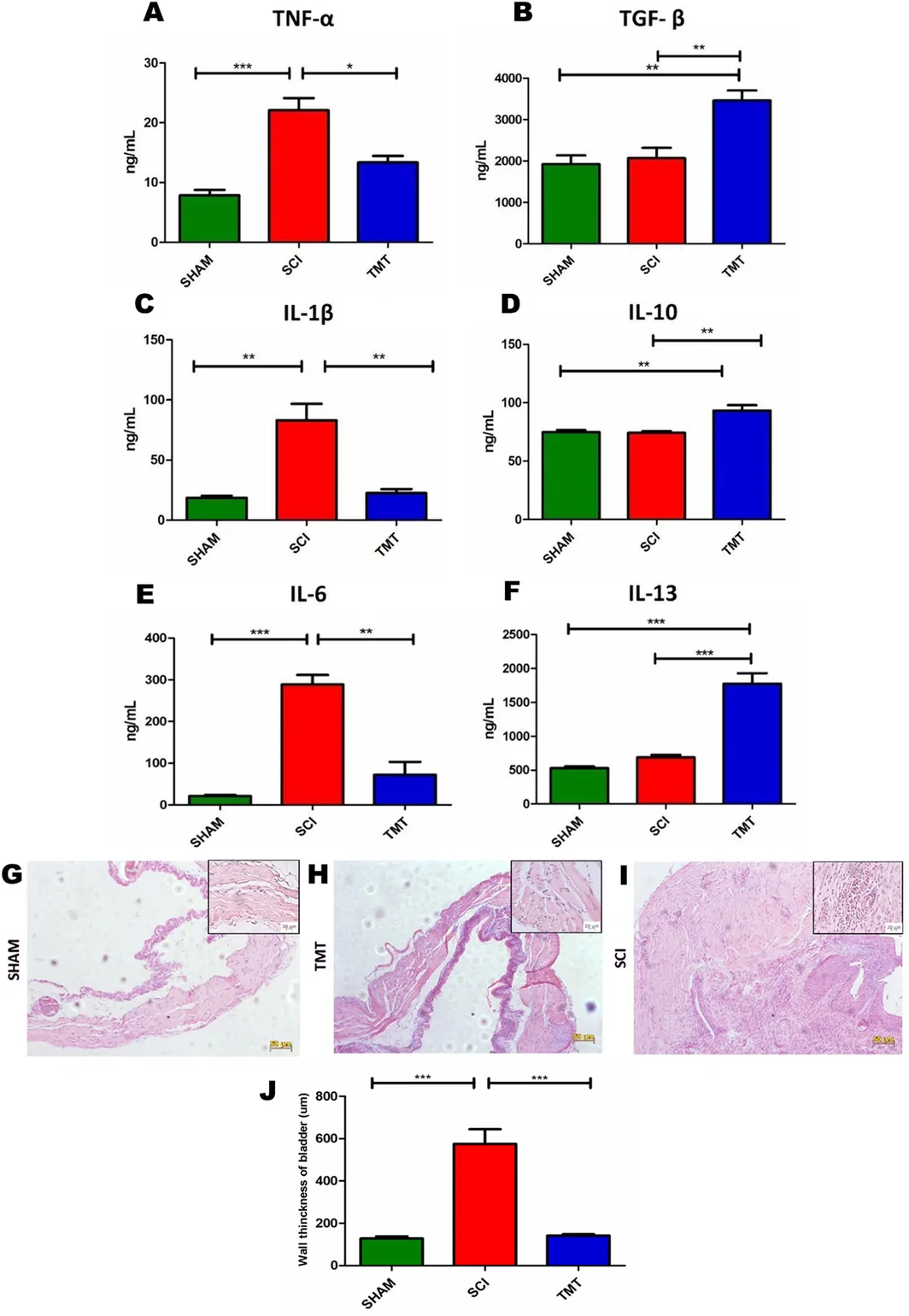
Treadmill exercise helps the modulation of the inflammatory microenvironment to a more anti‐inflammatory and functional profile after spinal cord injury. (A, C, E) TNF‐α; TGF‐β1; IL‐1β. (B, D, F) IL‐10, IL‐6 and IL‐13, in ng/mL. Among the cytokines, the TMT group stands out, demonstrating better levels of anti‐inflammatory cytokines. (G–I) Qualitative analysis of the bladder wall, per 50 μm scale group, 20×. (J) Bar graph representing bladder thickness. A significant difference was found between the SCI group in relation to SHAM and TMT. Statistical analysis: One‐way ANOVA and Tukey's post‐test. Values: *p < 0.05; **p < 0.01 and ***p < 0.01. *n* = 3/group.

To evaluate the relationship between spinal cord injury, systemic dysfunction and inflammatory repercussions, we analysed the thickness of the bladder wall. The qualitative (Figure 4G–I) and quantitative (Figure 4J) assessments showed that the TMT group (141.577 ± 0.33) had bladder wall thickness very close to the normal pattern observed in the SHAM group (127.857 ± 0.46). Meanwhile, the SCI group (574.963 ± 1.92) showed significant hypertrophy of the muscular layer and an important inflammatory infiltrate, mostly mononuclear, with poor polymorphonuclear infiltration. The SHAM and TMT groups obtained a statistically significant difference, with better performance to the SCI (*p* < 0.001), but not between them.

To analyse the inflammation profile of the lesion microenvironment, we performed and evaluated immunostaining for TMEM119, Arginase, iNOS, GFAP and cleaved Caspase‐3. Firstly, we observed that cross‐sections labelled with TMEM119 and Arginase showed higher colocalisation in the TMT group. This finding was supported by Pearson's correlation analysis, which revealed a strong correlation (0.85 ± 0.66), suggesting that the majority of TMEM119‐positive microglia exhibit an M2‐like, anti‐inflammatory profile. In contrast, the SCI group showed a lower correlation (0.46 ± 0.46) (Figure 5A–J). This data infers the possibility of exercise as a therapy to modulate the inflammatory microenvironment, helping to polarise an anti‐inflammatory microglia profile. In relation to TMEM+iNOS quantification, the SCI group obtained a higher correlation between markers (0.836 ± 0.43), demonstrating a greater pro‐inflammatory population, with the prevalence of the M2 profile. In contrast, the TMT group (0.33 ± 0.49) showed a lower correlation, demonstrating beneficial effects of exercise in relation to the modulation of the pro‐inflammatory profile (Figure 5K–T).

**FIGURE 5:**
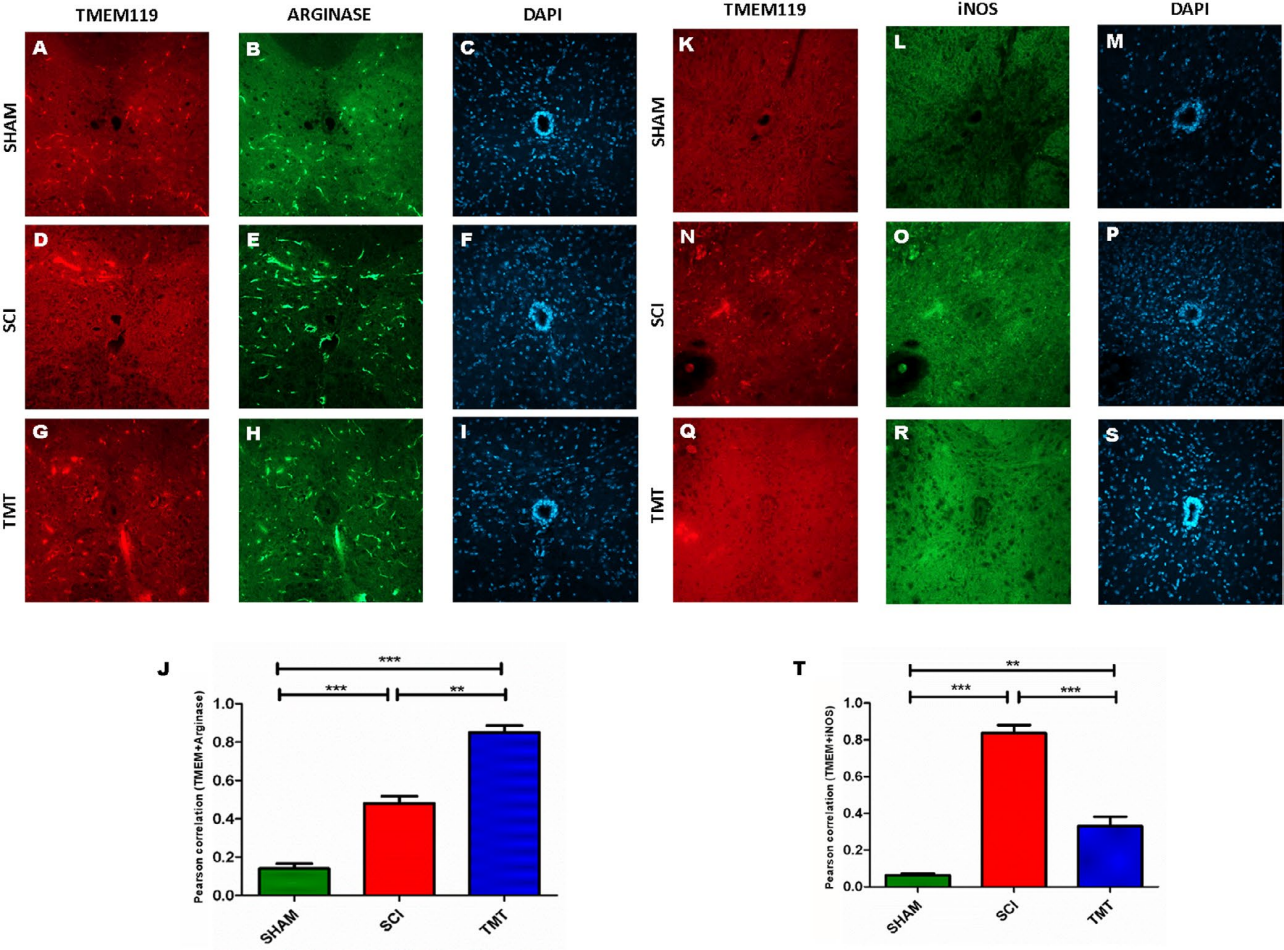
Microglia polarisation towards the M2 profile can be influenced by treadmill exercise. (A, D, G) Stainings for TMEM119 (general microglia population marker). (B, E, H) Stainings for Arginase (M2 profile microglial population marker), by Pearson's correlation. (J) The increased ratio of the general microglia population to the M2 profile in the TMT group stands out. (K, N, Q) Stainings for TMEM119 (general microglia population marker). (L, O, R) Markings for iNOS (M1 profile microglial population marker), by Pearson correlation. (T) The increased ratio of the general microglia population to the M1 profile in the SCI group stands out. C, F, I, M, P and S correspond to sections stained by DAPI. Statistical analysis: One‐way ANOVA and Tukey's post‐test. Values: **p < 0.01 and ***p < 0.01. *n* = 3/group.

In order to analyse the behaviour of another important glial cell, the astrocyte, within the context of its participation in the formation of the glial scar, labelling for GFAP was performed. In this context, the labelling of the TMT group was lower than that of the SCI group (*p* < 0.01) (Figure 6A–D), with the presence of a significant statistical difference between these groups, demonstrating that the exercise would induce a regulatory activity of reactive astrocytes, favouring a greater modulation of glial scar formation, aiding nervous plasticity.

**FIGURE 6:**
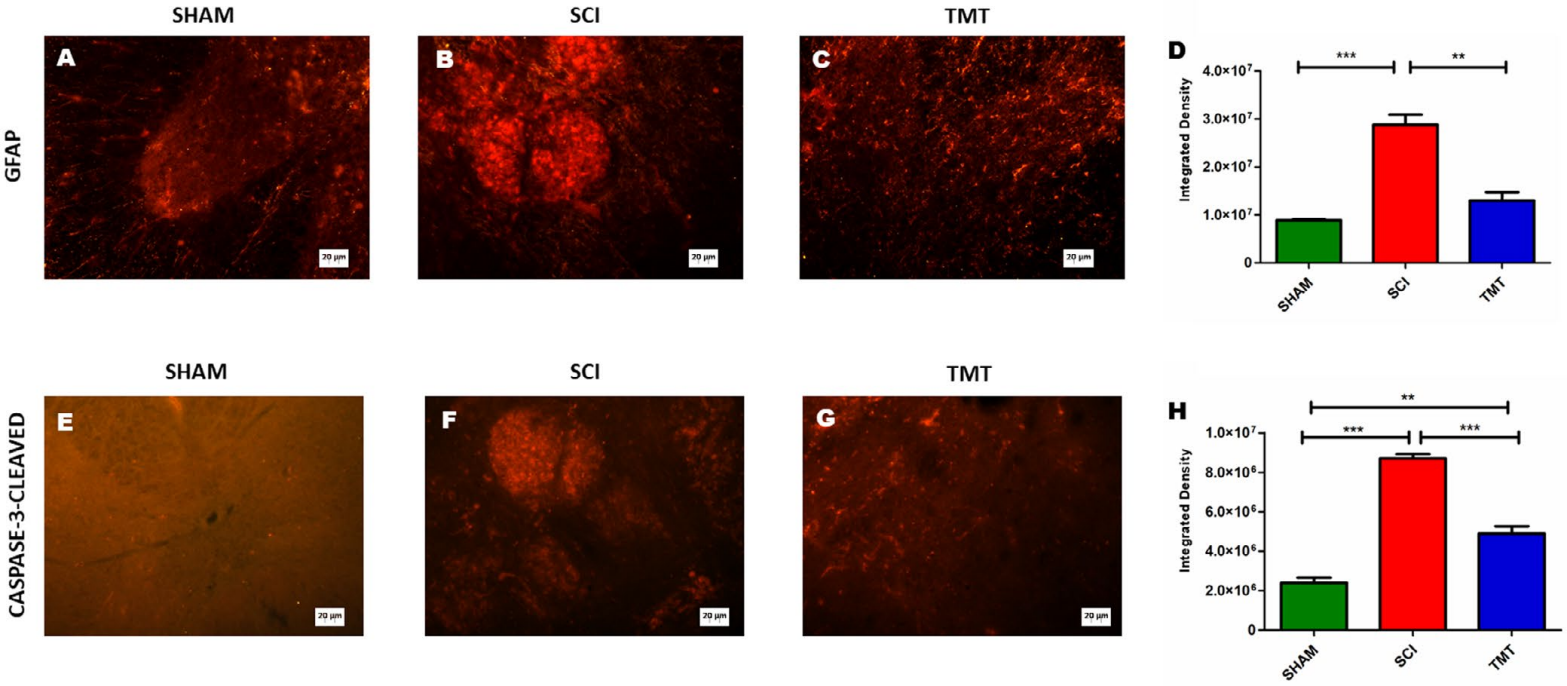
Glial scar formation and cell death regulation can be influenced by physical exercise. (A–C) The groups were evaluated, based on the integrated density, to analyse the area of GFAP labelling, in order to evaluate the area of glial scar formation. (D) The greater density of labelling for the SCI group stands out, in contrast to the TMT group. (E–G) The groups were evaluated, based on integrated density, to analyse the area of cleaved Caspase‐3‐labelling, in order to evaluate the area of glial scar formation. (H) The highest density of labelling for the SCI group stands out, in contrast to the TMT group, demonstrating a statistically significant difference between the groups. Statistical analysis: One‐way ANOVA and Tukey's post‐test. Values: **p < 0.01 and ***p < 0.01. *n* = 3/group.

In the context of cell survival, we assessed the level of cell death activation through labelling by cleaved caspase‐3, which is described as an enzyme that acts on apoptosis and is widely used to mark the activation of apoptotic activity. When evaluating the groups, we noticed a lower integrated density labelling in the TMT group (*p* > 0.001) when compared to the SCI group (Figure 6E–H), with the presence of a statistically significant difference between these groups. This data points to the benefits of exercise in relation to cell survival in the face of an injurious process, such as traumatic spinal cord injury.

We analysed the distribution of myelinated nerve fibres in the groups studied (Figure 7A–C). Qualitatively, the analysis revealed that the SCI group exhibited greater tissue disorganisation, the presence of small cavitations in the white matter and numerous degenerating nerve fibres when compared to the TMT group.

**FIGURE 7:**
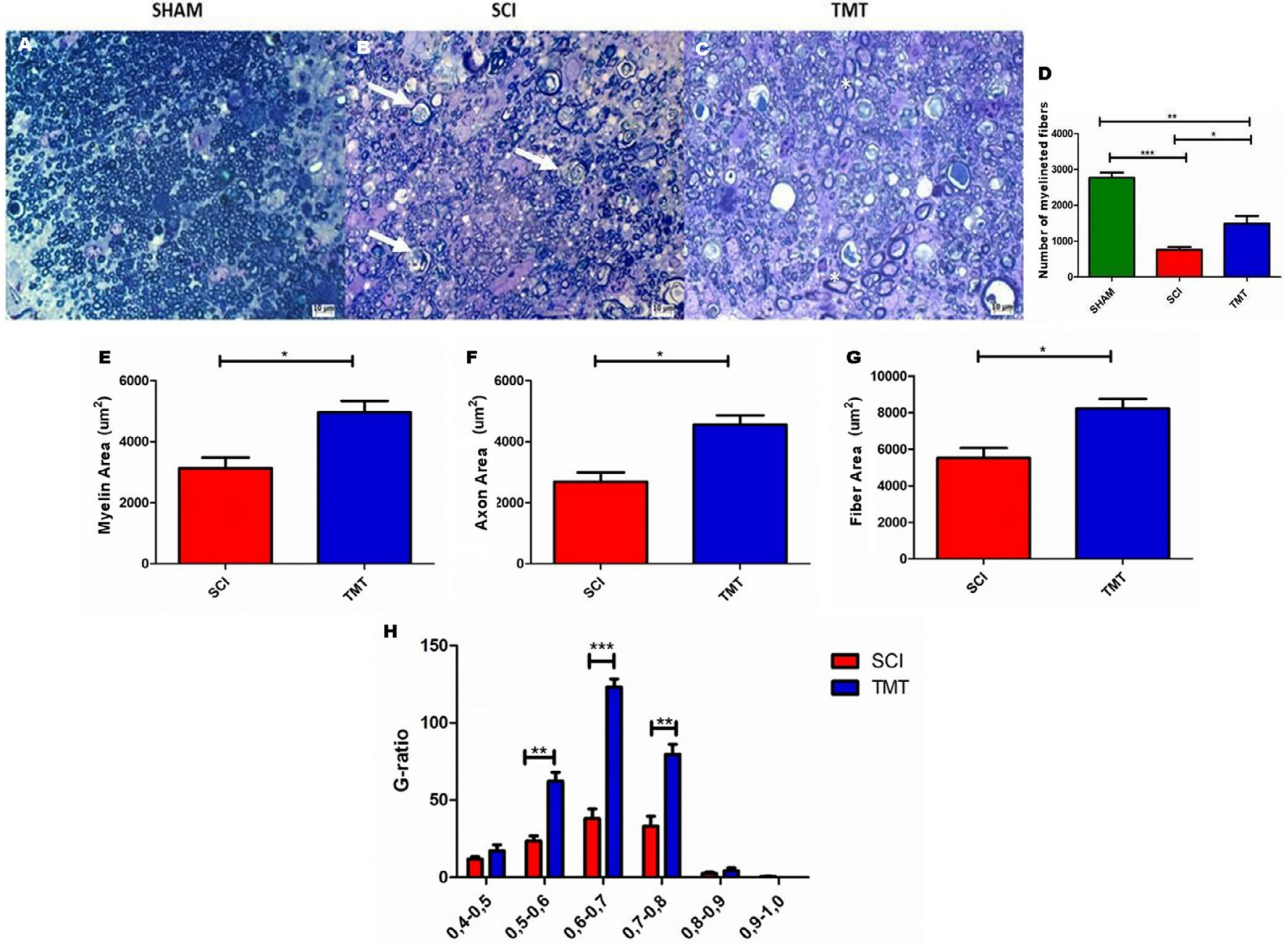
Treadmill training can influence nerve regeneration. (A–C) Semi‐thin cross‐sections stained with toluidine blue, named after the belonging group. SHAM group showing the typical organisation of the white matter area in the intact spinal cord (SM). Note several degenerating myelin fibres (arrows) and greater tissue disorganisation in the SCI group when compared to the TMT treated group, which exhibits preserved fibres of different calibres (asterisks). (D) Bar graph showing the average number of preserved myelin fibres per group. The treated groups obtained a greater quantity of preserved myelin fibres compared to controls. (E) Nerve fibre area; (F) myelin area; (G) axon area. (H) Stratification by range of G ratio values. All groups exhibited the majority of fibres in the range between 0.5–0.8 μm. The TMT group showed a shift in the graph to the left, showing more fibres in the range between 0.6 and 0.8. Scale Bar = 100 μm. Values: *p < 0.05, **p < 0.01; ***p < 0.001. Statistical Analysis: One‐way ANOVA and Tukey's Post‐test. *n* = 3/group.

The TMT groups presented a greater number of preserved myelinated fibres (Figure 7D) (1658 ± 73.58) when compared to the SCI group (199.7 ± 20.66, *p* < 0.05), 56 days after the injury. To analyse the g‐ratio, we quantified the myelin, fibre and axon areas with the ImageJ programme. Regarding the myelin area (Figure 7E), the TMT group (4963 ± 37.76, *p* < 0.05) showed a statistically significant difference when compared to the SCI group (3130 ± 36.63), delivering better performance. The TMT group presented higher values in terms of axon area compared to the SCI group alone (Figure 7F) (SCI 2694 ± 136.0 and TMT 4558 ± 111.3, *p* < 0.05). In Figure 7G, there was a difference between the analysed groups regarding the fibre area (SCI 5516 ± 136.0 and TMT 8223 ± 111.3, *p* < 0.05). Figure 7H shows the graph with the stratified g‐ratio results. The number of fibres in the range of 0.6–0.7 in the TMT group (123.2 ± 7.000, *p* < 0.001) was statistically different in relation to the SCI group (38 ± 8.667). In the range of 0.5–0.6 and 0.7–0.8, the TMT group demonstrated a statistically significant difference in relation to the SCI (*p* < 0.01).

Microtomography proved to be an important technique for evaluating the impact of this injury model on the destruction of spinal cord tissue and for observation of the regenerative response in three dimensions. We can see in Figure 8A–C, the 2D montage of the spinal cord and the cross‐sections of the rostral region (top), epicentre of the lesion (middle) and caudal region (base), in all groups at 56 days. Based on the organisation of tissues in cross‐sections, it can be qualitatively observed that the white matter architecture is more preserved in the TMT group when compared to the SCI group, showing an organisation closer to the SHAM group. Regarding the extent of the lesion area, the SCI group (0.382 ± 0.78; *p* < 0.05) presented a larger lesion area when compared to the TMT group (0.243 ± 0.65), which demonstrates that exercise has a potential protective capacity (Figure 8D–G).

**FIGURE 8:**
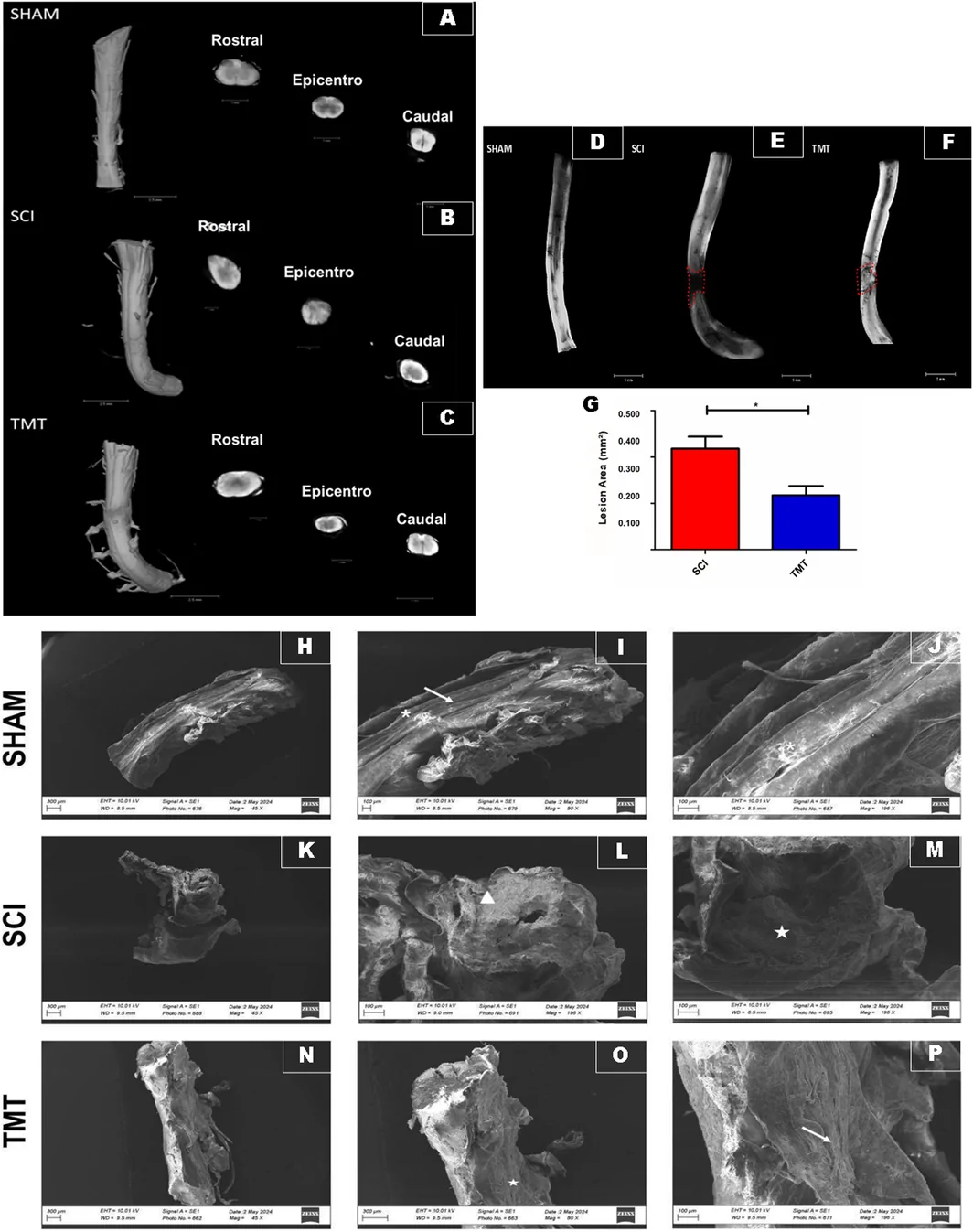
Exercise can influence the plasticity and microenvironment of spinal cord injury. (A–C) For each group, we can see the 2D model (on the left of the image) and the cross‐section (on the right of the image). Scale: 2.5 m (complete marrow) and 1 mm (cross section) (*n* = 3/group). (D–F) Micrograph of the SHAM, SCI and TMT groups. On this axis, we can observe the demarcation of the lesion area (red circle). Scale: 2.5 mm (cross section). (G) Bar graph to compare the lesion area (mm^2^) between SCI and TMT. A significant statistical difference was observed between the groups. (H–P) Scanning microscopy of the SHAM, SCI and TMT groups, 56 days after the injury. Scale of 300 μm (45× and 80×) and 100 μm (136×). Statistical analysis: One‐way ANOVA and Tukey's post‐test. *p < 0.05. *n* = 3/group.

To verify the general characteristics of the spinal topography after the injury, we evaluated the SHAM group, as a parameter of normality and the SCI and TMT groups (Figure 8H–P), at magnifications of 45×, 80× and 196×, respectively in each column. When we qualitatively analyse the material from the SHAM group, we can observe the orientation and organisation of the intact tracts (arrow), as well as the intact vasculature (asterisk). In the SCI group, it is possible to notice the disorganisation of the grey matter (arrowhead) and an important cavitary region at the site of the lesion (star). In the TMT group, the cavitary region appears, qualitatively, to have a smaller area, demonstrating greater tissue preservation and better visualisation of the tracts.

## 4 Discussion

The present study demonstrates that treadmill rehabilitation promotes functional recovery after compressive spinal cord injury (SCI) while reshaping the inflammatory microenvironment towards a pro‐regenerative state. Beyond improving locomotor performance, exercise attenuated neuroinflammation, promoted anti‐inflammatory microglial polarisation, reduced astrogliosis and apoptotic activity, preserved spinal cord architecture and myelinated fibres and improved motor conduction and neuromuscular integrity. Together, these findings indicate that the benefits of treadmill rehabilitation extend beyond motor training by modulating the secondary injury cascade and creating a permissive environment for tissue preservation and neural repair.

Behavioural analyses showed that treadmill‐trained animals achieved superior locomotor recovery, as demonstrated by higher BMS scores and improved Ladder Walking Test performance. These findings indicate enhanced gross locomotion and skilled motor coordination, corroborating previous reports that locomotor training promotes functional recovery during the subacute and chronic phases of SCI [17, 23]. Although daily rehabilitation protocols are frequently used, increasing evidence suggests that recovery depends on exercise intensity, timing, cumulative workload and recovery intervals rather than training frequency alone. Consistent with this concept, the protocol adopted in the present study, previously optimised by our group, effectively promoted functional recovery despite being performed only three times per week.

Electrophysiological findings further supported the behavioural improvements. Higher CMAP amplitudes and shorter latencies in treadmill‐trained animals indicate more efficient impulse conduction through preserved descending motor pathways. Although electrophysiological recordings do not directly demonstrate axonal regeneration, they provide objective functional evidence that complements the behavioural and morphological findings. The improved CMAP parameters were accompanied by greater preservation of myelinated fibres, more favourable g‐ratio values, reduced lesion extension and better maintenance of neuromuscular junctions, indicating that treadmill rehabilitation preserves neural conduction efficiency after SCI [24]. Although transvertebral stimulation may introduce greater variability than peripheral nerve stimulation, identical acquisition parameters were used for all groups and electrophysiological findings were interpreted together with independent behavioural, histological, ultrastructural and neuromuscular analyses.

Treadmill rehabilitation also attenuated muscle atrophy and preserved neuromuscular junction integrity, supporting maintenance of peripheral targets during spinal cord repair. Similar observations have been reported previously [23]. These effects are likely associated with exercise‐induced expression of neurotrophic factors, including BDNF, NT‐3 and GDNF, which promote neuronal survival, synaptic stability and neuromuscular plasticity [25, 26]. Consistent with these mechanisms, trained animals exhibited greater preservation of muscle mass, neuromuscular junction morphology, myelinated fibres, axonal area and a more favourable g‐ratio distribution, approaching the optimal range for efficient spinal cord conduction. Together with previous evidence demonstrating enhanced neuronal survival and adaptive plasticity after exercise [12, 20, 27], these findings suggest that treadmill rehabilitation preserves both central and peripheral components of the motor system.

A major finding of this study is that treadmill rehabilitation coordinately modulated the inflammatory microenvironment after SCI. Exercise reduced the expression of TNF‐α, IL‐1β and IL‐6 while increasing IL‐10, IL‐13 and TGF‐β, indicating a shift towards a pro‐regenerative inflammatory profile. During moderate exercise, skeletal muscle‐derived IL‐6 acts as a myokine, promoting anti‐inflammatory mediators such as IL‐10 while suppressing TNF‐α production [24, 28]. This response is particularly relevant after SCI, in which excessive cytokine production sustains microglial activation, immune‐cell infiltration and secondary tissue degeneration [29]. Furthermore, IL‐1β and IL‐6 stimulate iNOS activation, increasing nitric oxide production and contributing to demyelination and neuronal apoptosis [30].

Immunofluorescence analyses reinforced these findings by demonstrating increased TMEM119/Arginase‐1 and reduced TMEM119/iNOS colocalisation, indicating polarisation towards an anti‐inflammatory microglial phenotype. These changes were accompanied by reduced GFAP immunoreactivity and lower cleaved caspase‐3 expression, suggesting attenuation of reactive astrogliosis and apoptotic activity. Because persistent astrocyte activation contributes to glial scar formation and limits axonal regeneration [31, 32, 33], these findings support the concept that exercise modulates multiple components of the secondary injury cascade rather than isolated inflammatory pathways [13, 25].

The anti‐inflammatory effects of treadmill rehabilitation also extended beyond the spinal cord. Trained animals exhibited bladder morphology comparable to SHAM animals, whereas untreated SCI mice developed muscular hypertrophy and inflammatory remodelling. Although bladder function was not directly evaluated, these findings suggest attenuation of autonomic complications associated with SCI, which are major contributors to urinary tract infections, repeated hospitalisation and impaired quality of life [26, 34, 35, 36].

Finally, micro‐computed tomography and scanning electron microscopy confirmed greater preservation of spinal cord architecture in treadmill‐trained animals, including reduced lesion extension and improved organisation of grey and white matter. These findings agree with previous reports demonstrating adaptive remodelling of descending motor pathways after locomotor rehabilitation [37, 38]. Although neurotrophic factors were not directly quantified, the combined preservation of spinal cord organisation, myelinated fibres, neuromuscular junctions and reduced cleaved caspase‐3 expression is consistent with exercise‐induced neuroprotective mechanisms described previously [15, 39].

In terms of limitations, the exclusive use of female participants in this study was a deliberate methodological choice to reduce behavioural variability and increase data consistency. Furthermore, physiological issues related to spinal cord injury were considered, since the presence of male genitalia, even during testing, could worsen the condition during the recovery period, mainly due to dragging of the hind limbs. However, the inclusion of both sexes in future studies will be important to confirm the broader applicability of these findings.

Taken together, our findings demonstrate that treadmill rehabilitation should be regarded not only as a strategy to improve locomotor performance but also as a biological intervention capable of attenuating the secondary injury cascade after SCI. By integrating behavioural, electrophysiological, molecular, ultrastructural and imaging analyses, this study provides mechanistic evidence that appropriately dosed treadmill exercise promotes coordinated neuroprotection, preserves neural circuitry and creates a favourable microenvironment for tissue repair following compressive spinal cord injury.

## 5 Conclusion

Treadmill rehabilitation promoted functional recovery after compressive spinal cord injury by coordinating multiple neuroprotective mechanisms. Exercise shifted the inflammatory microenvironment towards a pro‐regenerative profile, reduced microglial and astrocytic reactivity, attenuated apoptotic signalling, preserved spinal cord tissue organisation, improved neural conduction and maintained neuromuscular integrity. Collectively, the integration of behavioural, electrophysiological, molecular, ultrastructural and imaging analyses demonstrates that appropriately dosed treadmill exercise mitigates secondary injury and creates a favourable environment for neural repair. These findings support treadmill rehabilitation as a feasible and clinically translatable therapeutic strategy for enhancing recovery after spinal cord injury.

## Author Contributions

**Anne Caroline Rodrigues dos Santos Cavalcante:** conceptualization, investigation, writing – original draft, methodology, visualization, writing – review and editing, formal analysis, data curation, validation. **Renata Pereira Laurindo:** investigation, methodology. **Luiza dos Santos Heringer:** investigation, methodology. **Anna Lecticia Martinez Martinez Toledo:** investigation, methodology. **Gabriela Sena Souza:** investigation, methodology. **Gabriel Fidalgo Queiroz da Silva:** investigation, methodology. **Ana Carolina Campos dos Santos:** investigation, methodology. **Morgana Teixeira Lima Castelo Branco:** investigation, methodology, supervision. **Ana Maria Blanco Martinez:** supervision, formal analysis, conceptualization, funding acquisition, methodology, writing – review and editing. **Suelen Adriani Marques:** conceptualization, funding acquisition, methodology, writing – review and editing, formal analysis, supervision.

## Funding

The authors have nothing to report.

## Conflicts of Interest

The authors declare no conflicts of interest.

## Data Availability

The datasets generated and/or analysed during the current study are available from the corresponding author upon reasonable request.

## References

[1] M. S. Æsøy , S.‐E. H. Solvang , M. Grønning , and T. Rekand , “Epidemiology of Persistent Iatrogenic Spinal Cord Injuries in Western Norway,” Brain and Behavior 6 (2016): e00522. 10.1002/brb3.522 PMC5064335 27781137

[2] T. Hagg and M. Oudega , “Degenerative and Spontaneous Regenerative Processes After Spinal Cord Injury,” Journal of Neurotrauma 23, no. 3–4 (2006): 264–280. 10.1089/neu.2006.23.263 16629615

[3] J. D. Houle and M. P. Côté , “Axon Regeneration and Exercise‐Dependent Plasticity After Spinal Cord Injury,” Annals of the New York Academy of Sciences 1279, no. 1 (2013): 154–163. 10.1111/nyas.12052 PMC3616327 23531013

[4] P. J. Reier , M. A. Lane , A. L. Behrman , and D. R. Howland , Spinal Cord: Repair and Rehabilitation (eLS, 2009).

[5] N. Sezer , S. Akkuş , and F. G. Uğurlu , “Chronic Complications of Spinal Cord Injury,” World Journal of Orthopedics 6, no. 1 (2015): 24–33. 10.5312/wjo.v6.i1.24 PMC4303787 25621208

[6] C. Rouanet , D. Reges , E. Rocha , V. Gagliardi , and G. S. Silva , “Traumatic Spinal Cord Injury: Current Concepts and Treatment Update,” Arquivos de Neuro‐Psiquiatria 75, no. 6 (2017): 387–393. 10.1590/0004-282X20170048 28658409

[7] R. C. Sterner and R. M. Sterner , “Immune Response Following Traumatic Spinal Cord Injury: Pathophysiology and Therapies,” Frontiers in Immunology 13 (2023): 1084101. 10.3389/fimmu.2022.1084101 PMC9853461 36685598

[8] M. G. Fehlings and R. Vawda , “Cellular Treatments for Spinal Cord Injury: The Time Is Right for Clinical Trials,” Neurotherapeutics 8, no. 4 (2011): 704–720. 10.1007/s13311-011-0076-7 PMC3210356 22002087

[9] B. S. Mietto , K. Mostacada , and A. M. B. Martinez , “Neurotrauma and Inflammation: CNS and PNS Responses,” Mediators of Inflammation 2015 (2015): 251204. 10.1155/2015/251204 PMC4397002 25918475

[10] H. Y. F. Yong , K. S. Rawji , S. Ghorbani , M. Xue , and V. W. Yong , “The Benefits of Neuroinflammation for the Repair of the Injured Central Nervous System,” Cellular & Molecular Immunology 16, no. 6 (2019): 540–546. 10.1038/s41423-019-0223-3 PMC6804643 30874626

[11] S. A. Martinez , N. D. Nguyen , E. Bailey , et al., “Multimodal Cortical and Subcortical Exercise Compared With Treadmill Training for Spinal Cord Injury,” PLoS One 13, no. 8 (2018): e0202130. 10.1371/journal.pone.0202130 PMC6084979 30092092

[12] D. Tang , X. Wang , Y. Chen , et al., “Treadmill Training Improves Respiratory Function in Rats After Spinal Cord Injury by Inhibiting the HMGB1/TLR‐4/NF‐κB Signaling Pathway,” Neuroscience Letters 782 (2022): 136686. 10.1016/j.neulet.2022.136686 35595191

[13] H. Tang , Y. Gu , L. Jiang , G. Zheng , Z. Pan , and X. Jiang , “The Role of Immune Cells and Associated Immunological Factors in the Immune Response to Spinal Cord Injury,” Frontiers in Immunology 13 (2023): 1070540. 10.3389/fimmu.2022.1070540 PMC9849245 36685599

[14] V. R. Edgerton , S. J. Kim , R. M. Ichiyama , Y. P. Gerasimenko , and R. R. Roy , “Rehabilitative Therapies After Spinal Cord Injury,” Journal of Neurotrauma 23, no. 3–4 (2006): 560–570. 10.1089/neu.2006.23.560 16629637

[15] H. R. Kobravi , A. Moghimi , and Z. Khodadadi , “Effect of Treadmill Training Protocols on Locomotion Recovery in Spinalized Rats,” Journal of Medical Signals and Sensors 7, no. 1 (2017): 53–57. PMC5394807 28487834

[16] M. H. Trimble , C. G. Kukulka , and A. L. Behrman , “The Effect of Treadmill Gait Training on Low‐Frequency Depression of the Soleus H‐Reflex: Comparison of a Spinal Cord Injured Man to Normal Subjects,” Neuroscience Letters 246, no. 3 (1998): 186–188. 10.1016/s0304-3940(98)00259-6 9792623

[17] A. C. R. D. Santos , R. P. Laurindo , F. M. Pestana , et al., “Exercise Volume Can Modulate the Regenerative Response to Spinal Cord Injury in Mice,” Neurotrauma Reports 5, no. 1 (2024): 721–737. 10.1089/neur.2024.0023 PMC11319863 39144452

[18] D. M. Basso , L. C. Fisher , A. J. Anderson , L. B. Jakeman , D. M. McTigue , and P. G. Popovich , “Basso Mouse Scale for Locomotion Detects Differences in Recovery After Spinal Cord Injury in Five Common Mouse Strains,” Journal of Neurotrauma 23, no. 5 (2006): 635–659. 10.1089/neu.2006.23.635 16689667

[19] G. A. Metz and I. Q. Whishaw , “The Ladder Rung Walking Task: A Scoring System and Its Practical Application,” Journal of Visualized Experiments 28 (2009): 1204. 10.3791/1204 PMC2796662 19525918

[20] T. B. Massoto , A. C. R. Santos , B. S. Ramalho , F. M. Almeida , A. M. B. Martinez , and S. A. Marques , “Mesenchymal Stem Cells and Treadmill Training Enhance Function and Promote Tissue Preservation After Spinal Cord Injury,” Brain Research 1726 (2020): 146494. 10.1016/j.brainres.2019.146494 31586628

[21] D. F. P. A. Durço , F. M. Pestana , J. T. Oliveira , et al., “Grafts of Human Adipose‐Derived Stem Cells Into a Biodegradable Poly (Acid Lactic) Conduit Enhances Sciatic Nerve Regeneration,” Brain Research 1747 (2020): 147026. 10.1016/j.brainres.2020.147026 32750328

[22] J. Albers , M. A. Markus , F. Alves , and C. Dullin , “X‐Ray Based Virtual Histology Allows Guided Sectioning of Heavy Ion Stained Murine Lungs for Histological Analysis,” Scientific Reports 8, no. 1 (2018): 7712. 10.1038/s41598-018-26086-0 PMC5955938 29769600

[23] M. R. Marques , F. C. Nicola , E. F. Sanches , et al., “Locomotor Training Promotes Time‐Dependent Functional Recovery After Experimental Spinal Cord Contusion,” Neuroscience 392 (2018): 258–269. 10.1016/j.neuroscience.2018.08.033 30195056

[24] P. J. Ward , A. N. Herrity , S. J. Harkema , and C. H. Hubscher , “Training‐Induced Functional Gains Following SCI,” Neural Plasticity 2016 (2016): 4307694. 10.1155/2016/4307694 PMC4926009 27403345

[25] G. Brazg , M. Fahey , C. L. Holleran , et al., “Effects of Training Intensity on Locomotor Performance in Individuals With Chronic Spinal Cord Injury: A Randomized Crossover Study,” Neurorehabilitation and Neural Repair 31, no. 10–11 (2017): 944–954. 10.1177/1545968317731538 PMC5729047 29081250

[26] S. Cobianchi , A. Arbat‐Plana , V. M. Lopez‐Alvarez , and X. Navarro , “Neuroprotective Effects of Exercise Treatments After Injury: The Dual Role of Neurotrophic Factors,” Current Neuropharmacology 15, no. 4 (2017): 495–518. 10.2174/1570159X14666160330105132 PMC5543672 27026050

[27] E. Carro , J. L. Trejo , S. Busiguina , and I. Torres‐Aleman , “Circulating Insulin‐Like Growth Factor I Mediates the Protective Effects of Physical Exercise Against Brain Insults of Different Etiology and Anatomy,” Journal of Neuroscience 21, no. 15 (2001): 5678–5684. 10.1523/JNEUROSCI.21-15-05678.2001 PMC6762673 11466439

[28] A. Steensberg , C. P. Fischer , C. Keller , K. Møller , and B. K. Pedersen , “IL‐6 Enhances Plasma IL‐1ra, IL‐10, and Cortisol in Humans,” American Journal of Physiology. Endocrinology and Metabolism 285, no. 2 (2003): E433–E437. 10.1152/ajpendo.00074.2003 12857678

[29] D. J. Hellenbrand , C. M. Quinn , Z. J. Piper , C. N. Morehouse , J. A. Fixel , and A. S. Hanna , “Inflammation After Spinal Cord Injury: A Review of the Critical Timeline of Signaling Cues and Cellular Infiltration,” Journal of Neuroinflammation 18, no. 1 (2021): 284. 10.1186/s12974-021-02337-2 PMC8653609 34876174

[30] M. A. Anwar , T. S. Al Shehabi , and A. H. Eid , “Inflammogenesis of Secondary Spinal Cord Injury,” Frontiers in Cellular Neuroscience 10 (2016): 98. 10.3389/fncel.2016.00098 PMC4829593 27147970

[31] K. Alka , G. Mohammad , and R. A. Kowluru , “Regulation of Serine Palmitoyl‐Transferase and Rac1‐Nox2 Signaling in Diabetic Retinopathy,” Scientific Reports 12, no. 1 (2022): 16740, 10.1038/s41598-022-20243-2. PMC9537524 36202842

[32] T. Clifford , Z. Finkel , B. Rodriguez , A. Joseph , and L. Cai , “Current Advancements in Spinal Cord Injury Research—Glial Scar Formation and Neural Regeneration,” Cells 12 (2023): 853. 10.3390/cells12060853 PMC10046908 36980193

[33] S. Okada , M. Hara , K. Kobayakawa , Y. Matsumoto , and Y. Nakashima , “Astrocyte Reactivity and Astrogliosis After Spinal Cord Injury,” Neuroscience Research 126 (2018): 39–43. 10.1016/j.neures.2017.10.004 29054466

[34] M. J. Eckert and M. J. Martin , “Trauma: Spinal Cord Injury,” Surgical Clinics 97, no. 5 (2017): 1031–1045. 10.1016/j.suc.2017.06.008 28958356

[35] M. A. Fitzpatrick and N. Nwafo , “Urinary Tract Infection Diagnostic and Management Considerations in People With Spinal Cord Injury and Neurogenic Bladder,” Infectious Disease Clinics of North America 38, no. 2 (2024): 381–393. 10.1016/j.idc.2024.03.012 38580574

[36] P. E. Ludwig , A. A. Patil , A. J. Chamczuk , and D. K. Agrawal , “Hormonal Therapy in Traumatic Spinal Cord Injury,” American Journal of Translational Research 9, no. 9 (2017): 3881–3895. PMC5622236 28979667

[37] Y. Cao , Y. Zhou , S. Ni , et al., “Three Dimensional Quantification of Microarchitecture and Vessel Regeneration by Synchrotron Radiation Microcomputed Tomography in a Rat Model of Spinal Cord Injury,” Journal of Neurotrauma 34, no. 6 (2017): 1187–1199, 10.1089/neu.2016.4697. 27676128

[38] G. Sena , A. X. da Silva , L. P. Nogueira , et al., “Investigation of *Rhodnius prolixus* Head Anatomy Using Microtomography,” Journal of Microscopy 290, no. 3 (2023): 161–167. 10.1111/jmi.13186 37038805

[39] G. L. Chen , K. Sun , X. Z. Liu , et al., “Inhibiting Tau Protein Improves the Recovery of Spinal Cord Injury in Rats by Alleviating Neuroinflammation and Oxidative Stress,” Neural Regeneration Research 18, no. 8 (2023): 1834–1840. 10.4103/1673-5374.363183 PMC10154485 36751813

